# Oxidative Stress Biomarkers and Peripheral Endothelial Dysfunction in Rheumatoid Arthritis: A Monocentric Cross-Sectional Case-Control Study

**DOI:** 10.3390/molecules25173855

**Published:** 2020-08-25

**Authors:** Stefania Bassu, Angelo Zinellu, Salvatore Sotgia, Arduino Aleksander Mangoni, Alberto Floris, Giuseppina Farina, Giuseppe Passiu, Ciriaco Carru, Gian Luca Erre

**Affiliations:** 1 Dipartimento di Scienze Biomediche, Università degli Studi di Sassari, 07100 Sassari, Italy; bassustefania@tiscali.it (S.B.); azinellu@uniss.it (A.Z.); ssotgia@uniss.it (S.S.); giusyfary92@libero.it (G.F.); carru@uniss.it (C.C.); 2 Discipline of Clinical Pharmacology, College of Medicine and Public Health, Flinders University and Flinders Medical Centre, Adelaide 5001, Australia; arduino.mangoni@flinders.edu.au; 3 Medizinische Fakultät Carl Gustav Carus, Technische Universität Dresden, 01069 Dresden, Germany; 4 Rheumatology Unit, University Clinic and AOU of Cagliari, 09100 Cagliari, Italy; alberto.floris@unica.it; 5 Dipartimento di Scienze Mediche, Chirurgiche e Sperimentali, Università degli Studi di Sassari, 07100 Sassari, Italy; gpassiu@uniss.it; 6 Dipartmento di Specialità Mediche, UOC di Reumatologia, Azienda Ospedaliero-Universitaria di Sassari, 07100 Sassari, Italy; 7 Dipartimento di Scienze Biomediche, Azienda Ospedaliero-Universitaria di Sassari, 07100 Sassari, Italy

**Keywords:** rheumatoid arthritis, oxidative stress, biomarkers, proteins-SH, paroxonase-1, malondialdehyde, endothelial dysfunction, flow-mediated pulse amplitude tonometry, cardiovascular disease

## Abstract

Previous studies have suggested that oxidative stress may heighten atherosclerotic burden in rheumatoid arthritis (RA), but direct evidence is lacking. Objective: To evaluate the relationship between established plasma oxidative stress biomarkers and peripheral endothelial dysfunction (ED), a marker of early atherosclerosis, in RA. Methods: Paroxonase-1 (PON-1), protein-SH (PSH), and malondialdehyde (MDA) were measured in 164 RA patient s and 100 age- and sex-matched healthy controls without previous cardiovascular events. Peripheral ED, evaluated by flow-mediated pulse amplitude tonometry, was defined by log-transformed reactive hyperemia index (Ln-RHI) values < 0.51. Results: PON-1 activity and PSH concentrations were significantly reduced in RA patients compared to controls. In regression analysis, increased plasma MDA levels were significantly associated with reduced Ln-RHI [B coefficient (95% CI) = −0.003 (−0.005 to −0.0008), *p* = 0.008] and the presence of peripheral ED (OR (95% CI) = 1.75 (1.06–2.88), *p* = 0.028). Contrary to our expectations, increased PON-1 activity was significantly associated, albeit weakly, with the presence of ED (OR (95% CI) = 1.00 (1.00–1.01), *p* = 0.017). Conclusions: In this first evidence of a link between oxidative stress and markers of atherosclerosis, MDA and PON-1 showed opposite associations with peripheral vasodilatory capacity and the presence of ED in RA. Further studies are needed to determine whether this association predicts atherosclerotic events in the RA population.

## 1. Introduction

Rheumatoid arthritis (RA), is an autoimmune disease characterized by chronic systemic and articular inflammation, bone erosions and increased risk of all-cause and cardiovascular mortality [1]. Beside musculoskeletal features, RA is also characterized by systemic complications, including atherosclerotic cardiovascular disease, that are linked to the chronic systemic inflammatory state and the dysregulated immune response [2,3].

There is good evidence that RA patients develop early endothelial dysfunction (ED), accelerated arterial wall stiffening and increased plaque burden, features of atherosclerotic disease [4,5], predisposing them to fatal cardiovascular events and sudden death [3,6]. However, ED and increased atherosclerotic cardiovascular disease in RA are not fully accounted for by traditional cardiovascular risk factors, suggesting that other mechanisms are likely to be involved [7,8].

Among those mechanisms, oxidative stress has been linked to functional and structural cardiovascular alterations in animal models of chronic arthritis and in patients with RA, suggesting a complex interplay between oxidative stress, autoimmune response and inflammation in the development of atherosclerotic cardiovascular disease in RA [9]. Reactive oxygen species and reactive nitrogen species are highly reactive chemical compounds that have the potential to damage lipids, proteins and DNA favoring expression of neoantigens and initiation of autoimmunity in predisposed individuals. Accordingly, exaggerated reactive oxygen species formation and increased levels of markers of protein and lipid oxidation have been reported in several systemic autoimmune diseases, including RA [10,11,12,13,14,15,16].

Paroxonase-1 (PON-1) is a calcium-dependent High Density Lipoprotein (HDL)-associated esterase, that was shown to protect HDL and LDL from oxidation [17]. Moreover, PON-1 reduces foam cell and plaque formation through the inhibition of monocytes differentiation into macrophages [18]. Based on these activities, PON-1 is considered to play a crucial anti-atherosclerotic role. This proposition is further supported by the observation that a decreased PON-1 enzyme activity has been linked to an increased risk of cardiovascular disease [19]. In a prospective large cohort study of patients undergoing coronary angiography, those with the lowest PON-1 activity had 3.4 times greater hazard of major cardiovascular events compared to those with the highest PON-1 activity [20]. Although impaired PON-1 activity has been reported in RA patients [21,22,23], whether reduced PON-1 activity could explain, at least in part, the excess atherosclerotic burden in RA is unknown.

Plasma protein thiols (PSH) refers to the total amount of protein thiols in plasma, with the single free cysteinyl thiol of albumin Cys34 accounting for about 80% of reduced protein thiols [24]. PSH act as scavenger of reactive oxygen species, thus in the presence of exaggerated oxidative stress, the free -SH group of proteins may be oxidized, resulting in a decrease in PSH measurable groups [24]. Of note, depletion of plasma thiols has been reported to be associated to various surrogate measures of cardiovascular disease [25,26,27]. A significant reduction of plasma PSH has been demonstrated in RA patients compared to controls [28,29], but the involvement of plasma PSH depletion in the onset and progression of atherosclerosis in RA is still unexplored.

Malondialdehyde (MDA), a by-product of lipid peroxidation, is a validated biomarker of oxidative stress. MDA can spontaneously break down to form acetaldehyde (AA) and, together with AA, has been demonstrated to modify proteins to produce an MDA-AA protein adduct, termed malondialdehyde-acetaldehyde (MAA) [30]. Of note, plasma MDA concentrations in RA patients are increased compared to the general population [31] and increasing evidence suggests that MAA protein adducts and anti-MAA immune responses could play a pathogenic role in RA [32,33]. MDA and MAA have been linked to accelerated atherosclerosis and increased risk of cardiovascular disease in the general population [34,35,36,37]. However, no evidence is available about the role of MDA (and MAA and anti-MAA) in the increased burden of atherosclerosis in RA.

As previously discussed, PON-1, PSH, and MDA are linked to surrogate measures of cardiovascular disease in the general population; however, their association with markers of atherosclerosis, e.g., peripheral ED, in RA is unknown. Therefore, in this study we evaluated the relationship between established markers of oxidative stress and peripheral ED in RA patients free from previous cardiovascular events. Levels of these oxidative stress biomarkers were also evaluated in healthy controls (controls).

## 2. Results

### 2.1. Patients and Controls

The study included 164 patients with RA (62 males/102 females; mean age 55.0 ± 6.8 years) and 101 controls (50 males/51 females; mean age 54.9  ±  5.6 years). According to protocol, the age, the gender distribution, and the prevalence of cardiovascular risk factors across groups were similar (Table 1), barring the mean concentration of triglycerides that was significantly higher in RA patients than in controls (Table 1). RA patients included in the study had long-standing disease with moderate activity (Table 2).

### 2.2. Oxidative Stress Biomarkers, Ln-RHI and Peripheral ED

Multiple comparisons by ANOVA showed significantly lower plasma PSH concentrations and PON-1 activity in RA patients compared to controls (3.15 ± 3.7 vs. 3.77 ± 0.7 µmol/gr protein, *p* < 0.001, and 109.73 ± 67.4 vs. 128.09 ± 76.2 U/L, *p* = 0.042, respectively) (Table 3). In bivariate correlation analysis in RA patients, there was a significant negative correlation between higher plasma MDA concentrations and peripheral vasodilatory capacity (Pearson’s correlation −0.17, *p* = 0.025) (Table 4). We also found a significant inverse correlation between MDA and PSH and ESR (Pearson’s correlation −0.21, *p* = 0.013 for MDA; Pearson’s correlation −0.19, *p* = 0.019 for PSH) (Table 4).

In regression analysis adjusted for age, gender and ESR, MDA demonstrated a significantly negative correlation with Ln-RHI, suggesting that higher plasma MDA concentrations were associated with impaired peripheral vasodilatory capacity in RA (Table 5).

Accordingly, in logistic regression higher plasma MDA concentrations were significantly and independently associated to the presence of ED (OR (95% CI) = 1.75 (1.06–2.88), *p* = 0.028) (Table 6). However, contrary to our expectations, increased but not reduced PON-activity was significantly associated, albeit weakly, with the presence of peripheral ED (OR (95% CI) = 1.00 (1.00–1.01)) (Table 6).

## 3. Discussion

Recent studies suggest the involvement of oxidative stress in the pathogenesis of accelerated atherogenesis and increased cardiovascular disease in RA [9]. Therefore, in this study we assessed (i) plasma concentrations of PON-1, PSH, and MDA in RA compared with controls, and (ii) the relationship of these biomarkers with peripheral vasodilatory capacity and the presence of peripheral ED in RA patients.

There is good evidence that increased HDL cholesterol concentrations are inversely associated, albeit in a nonlinear relationship, with the risk of future cardiovascular events in the general population [38]. Promotion of cholesterol efflux, inhibition of LDL oxidation, neutralization of ox-LDL inflammatory effect on arterial wall and atherosclerotic plaque evolution, are among the mechanisms proposed to explain the cardiovascular protective effects of HDL cholesterol. PON-1 is an HDL-associated enzyme that protect LDL from oxidation [17]. Previous studies have suggested that PON-1 activity is impaired in RA patients as compared to healthy controls [22,23]. In line with these studies, we found a significant reduction of PON-1 activity in RA patients compared to controls.

Different mechanisms have been suggested to explicate the impairment of PON-1 activity in RA patients, including the enhanced generation of reactive oxygen species [39] compositional changes of HDL [23], inhibitory effect of proinflammatory cytokines on the liver synthesis of PON-1 [40], and genetic polymorphisms of the PON-1 gene [41,42].

However, the effect of proinflammatory markers on PON-1 activity is still debated, with some studies reporting repression, while others suggesting upregulation of PON-1 gene expression and activity [40,43]. Accordingly, in our study, PON-1 activity was not significantly correlated with markers of systemic inflammation.

A recent meta-analysis of 12 studies reported a significant association between PON-1 polymorphisms and enzyme activity in RA [44]. A relationship between PON-1 polymorphisms, PON-1 activity, atherogenic lipid profile, and atherosclerotic plaque burden has also been reported, suggesting that the genetic regulation of PON-1 activity may contribute to the increased cardiovascular burden in RA [21,42,45]. While impaired PON-1 activity and increased concentrations of oxidized LDL have been involved in the development of ED [46] the evidence supporting this link in RA patients is currently limited.

Kerekes G et al. [43] found no association between surrogate measures of ED and atherosclerosis and levels of PON-1 activity in RA. In our study we demonstrate a paradoxical significant relationship between better PON-1 activity, suggesting lower oxidative stress burden, and the presence of peripheral ED in RA patients. Therefore, at the moment, the direction and the magnitude of the relationship between PON-1 activity and endothelial function are unclear, suggesting that other factors besides PON-1 activity and HDL cholesterol composition and function are involved.

A significant depletion of plasma thiols, including PSH, has been reported in the context of acute cardiovascular events and ventricular dysfunction in the general population [25,26,27]. Moreover, a significant independent relationship between plasma/serum transsulfuration pathway, thiol concentrations and cardiovascular risk scores has been observed at the population level [47]. Of note, in line with previous reports [28,29], we demonstrated a significant reduction of plasma PSH concentrations in RA patients compared to controls.

However, little knowledge is currently available on whether there are independent associations between PSH and surrogate markers of cardiovascular disease in RA. Therefore, we explored this issue evaluating the relationship between PSH and peripheral endothelial dysfunction. However, we did not find any significant association between plasma PSH level and peripheral vasodilatory capacity and the presence of peripheral ED in our series of RA patients.

MDA and MAA have been associated to early atherosclerotic process and increased risk of cardiovascular events in the general population [34,35,36,37]. Of note, increased plasma MDA concentrations in RA patients compared to the general population were reported [31], supporting the hypothesis that raised levels of MDA may be linked to the increased burden of atherosclerotic cardiovascular disease in RA. In this study, we found no significant differences in plasma MDA concentrations between RA patients and controls. However, for the first time, we demonstrated a significant independent association between higher plasma MDA levels and lower peripheral vasodilatory capacity and the presence of peripheral ED in RA. Pending further studies on the role of MDA in the development of peripheral ED, this result may suggest the involvement of increased lipid peroxidation in the pathogenesis of increased cardiovascular burden in the RA population.

Collectively taken, these data, in the context of a documented increase in oxidative stress in the RA population, show opposite associations of established oxidative stress biomarkers with microvascular vasodilation capacity and ED. Therefore, further studies are needed to confirm these associations, to explore the pathophysiology underlining them, and to address whether oxidative stress may act directly to modulate or, rather, is merely associated to specific factors involved in peripheral ED in RA patients.

The main limitation of this study was that RA patients were under treatment with immunosuppressors for the control of disease activity at the moment of biomarkers and PAT evaluation. Moreover, the cross-sectional design of this study did not enable us to make conclusive considerations about relationship between oxidative stress and microvascular function in RA. Moreover, the evaluation of additional biomarkers of oxidative stress (e.g., activity of superoxide dismutase and catalase, concentrations of vitamin C, vitamin E, zinc, copper and uric acid) might provide further mechanistic insights into the relationship between oxidative stress and peripheral ED in RA”.

## 4. Materials and Methods

### 4.1. Subjects

We studied 164 RA patients (mean age 55.4 ± 6.8 years, range 45–85), classified according to 2010 EULAR/ACR criteria [48], without history of previous cardiovascular events, prospectively enrolled in the Bio-RA study (Evaluation of new BIO-markers of atherosclerosis in Rheumatoid Arthritis) between October 2015 and July 2017. The Bio-RA study is an ancillary study of the Endothelial Dysfunction Evaluation for Coronary Heart Disease Risk Estimation in Rheumatoid Arthritis study (EDRA study; ClinicalTrials.gov: NCT02341066). Inclusion and exclusion criteria of the Bio-RA and EDRA studies were recently published [7].

One hundred and one healthy controls (controls) (mean age 54.9 ± 5.6 years), matched for age, gender and cardiovascular risk factors, attending the blood donor bank of the Azienda Ospedaliero-Universitaria of Sassari (Italy) were enrolled in the study. The Bio-RA and the EDRA studies were approved by the Ethics Committee of Azienda ASL 1 of Sassari (Italy) (2126/CE-2015 and 2219/CE-2015) and conducted in accordance with the Declaration of Helsinki. Written informed consent was obtained from each subject before the study.

### 4.2. Clinical Variables

The following data were collected in all participants of the Bio-RA and EDRA studies: hypertension (defined as a blood pressure ≥140/90 mmHg or treatment with antihypertensive medications), diabetes (diagnosed according to the patient’s history and/or treatment with insulin or oral hypoglycemic agents), dyslipidemia (defined according to either a recent lipid profile, the patient’s history and/or treatment with hypolipidemic drugs) and current smoking habit. In RA patients, the following data were also collected: current treatment with steroids or synthetic or biological disease-modifying antirheumatic drugs (sDMARDs); current treatment with Tumor Necrosis Factor (TNF) inhibitors (TNFi); C-reactive protein (CRP) concentrations; erythrocyte sedimentation rate (ESR); Disease Activity Score-28 (DAS-28); positivity for Rheumatoid Factor (RF) and anticitrullinated cyclic peptide antibodies (ACPA).

### 4.3. Laboratory Variables

Blood samples were collected using blood evacuation tubes containing EDTA (Vacutainer Systems Europe; Becton Dickinson, Meylan Cedex, France). Immediately after recovery, blood samples were centrifuged at 1500× *g* for 10 min, and plasma was removed and stored at −80 °C until assay. PON1 activity was determined using paraoxon (*O*,*O*-diethyl-*O*-*p*-nitrophenyl phosphate) as a substrate and measuring the increases in the absorbance at 412 nm due to the 4-nitro phenol formation [49,50]. The enzyme activity was computed from the molar extinction coefficient (17,000 M^−1^ cm^−1^) with a 1 nmol of 4-nitrophenol formed per minute used as an enzyme activity unit. PSH determination was performed spectrophotometrically at 405 nm using Ellman’s reagent (DTNB, 5,5′-dithiobis-2-nitrobenzoic acid) and a standard curve obtained using standard solutions of GSH. Lowry’s method was used to measure the plasma proteins amount [51] and this value was employed to normalize PSH levels. MDA and other aldehydes produced by lipid peroxidation induced by hydroxyl free radicals were measured by thiobarbituric acid reactive substances (TBARS) methodology according to the method described by Esterbauer and Cheeseman [52].

### 4.4. Flow-Mediated Pulse Amplitude Tonometry (PAT)

Fasting RA patients were studied in a quiet and temperature-controlled room. Two probes positioned on the middle finger of both hands, consisting of an expandable compartment, registered pulsatile volume changes occurring into the digital artery. Registered pulsatile volume changes were then recorded as pulse amplitude by the EndoPAT 2000 system (Itamar Medical Inc., Caesarea, Israel).

After a 5 min baseline period, the digital artery flow was interrupted by occluding the brachial artery after inflating a blood pressure cuff to suprasystolic pressures (60 mmHg above the baseline systolic blood pressure) for 5 min. Then, the digital pulse amplitude was registered after deflating the blood pressure cuff. The log-transformed ratio of the post-occlusion pulse amplitude signal compared with the baseline one was calculated and reported in standardized arbitrary units as Ln-RHI [53]: A Ln-RHI cutoff value <0.51 was used to define the presence of peripheral ED.

### 4.5. Statistical Analysis

Normality of data was assessed using the Kolmogorov–Smirnov test. Continuous variables were reported as mean values ± standard deviation (SD) or median values and interquartile range, as appropriate. Categorical variables were reported as frequencies (*n*) and percentages (%), as appropriate. Groups were compared using Student’s t-test, Mann–Whitney rank sum test, chi-squared test or Fisher exact test, as appropriate. Correlations analysis was performed by Pearson’s correlation or Spearman’s rank correlation, as appropriate. Multiple linear regression analysis was performed to evaluate the presence of a linear correlation between oxidative stress biomarkers and Ln-RHI. The variables related to endothelial dysfunction with a *p* < 0.05 at the univariate logistic regression analysis entered into a multivariate logistic regression model in which the “presence of endothelial dysfunction” was the variable to be explained. Results are expressed as the odds ratio (OR) and 95% confidence interval (95%CI). Analyses were performed using Stata 14 (StataCorp. 2015. Stata Statistical Software: Release 14. College Station, TX: StataCorp LP.). A *p* < 0.05 was considered statistically significant.

## 5. Conclusions

This study strengthens previous findings reporting an imbalance between oxidant and antioxidant factors in RA. Plasma MDA levels and PON-1 activity show opposite association with the presence of peripheral ED in RA. Therefore, further investigations are needed to clarify the role of oxidative stress markers, including MDA and PON-1, in the pathogenesis of peripheral ED and increased cardiovascular disease in RA patients.

## Figures and Tables

**Table 1:** Demographic characteristics and cardiovascular risk profile of rheumatoid arthritis (RA) patients and controls.

	Rheumatoid Arthritis *n* = 164	Controls *n* = 101	*p*
age, years	55.0 ± 6.8	54.9 ± 5.6	0.96
female, *n*(%)	102(62.2)	51(50.5)	0.07
hypertension, *n*(%)	47(28.7)	26(25.7)	0.639
dyslipidaemia, *n*(%)	36(21.9)	21(20.7)	0.855
diabetes, *n*(%)	9(5.4)	6(5.9)	0.684
current smoker, *n*(%)	46(28)	19(18.8)	0.098
total cholesterol, mg/dL	207.0 ± 36	212.1 ± 36	0.294
triglycerides, mg/dL	95.3 ± 45	82.3 ± 34	0.013
creatinine, mg/dL	0.82 ± 0.2	0.84 ± 0.1	0.567

**Table 2:** Rheumatoid arthritis specific features and cardiovascular parameters.

Variable	Value
disease duration, months	114.9 ± 99.8
DAS-28	3.4 ± 0.6
RF, %	65
CRP, mg/dL	2.9 ± 2.2
ESR, mm/h	27.6 ± 1.7
ACPA, %	62
steroid use, %	34
steroid, mg/day	2.4 ± 0.3
DMARDs use, *n*(%)	67
TNF-inhibitors use, *n*(%)	28
Ln-RHI	0.68 ± 0.02
endothelial dysfunction, %	24.3

Values are mean ± SD. ACPA, Anticitrullinated cyclic peptide antibodies; CRP, C-reactive protein; DAS-28, Disease Activity Score-28; DMARDs, disease modifying antirheumatic drugs; ESR, Erythrocyte Sedimentation Rate; Ln-RHI, Log-transformed reactive hyperemia index.

**Table 3:** Plasma oxidative stress biomarkers in rheumatoid arthritis patients vs. controls.

	Rheumatoid Arthritis *n* = 164	Controls *n* = 101	*p*
PON-1	109.73 ± 67.4	128.09 ± 76.2	0.042
PSH	3.15 ± 3.7	3.77 ± 0.7	<0.001
MDA	2.58 ± 0.7	2.52 ± 0.6	0.55

PON-1, paraoxonase-1; PSH, protein-SH; MDA, malondialdehyde.

**Table 4:** Correlation matrix between plasma oxidative stress biomarkers and RA features.

	PSH	PON-1	MDA	CRP	ESR	DAS28	Steroid	DMARDs	TNFi	Ln-RHI
PSH	1.0000									
PON-1	0.0812 0.3016	1.0000								
MDA	**0.1780** **0.0226**	0.1264 0.1067	1.0000							
CRP	−0.1265 0.1192	−0.0411 0.6135	−0.0039 0.9616	1.0000						
ESR	**−0.1901** **0.0190**	−0.1433 0.6135	**−0.2010** **0.0130**	**0.2349** **0.0037**	1.0000					
DAS28	−0.0937 0.2328	−0.0895 0.2543	−0.0898 0.2527	0.0186 0.8198	**0.6087** **0.0000**	1.0000				
steroid	−0.0073 0.9265	**−0.1590** **0.0433**	0.1208 0.1258	−0.0582 0.4781	**0.1756** **0.0310**	**0.1787** **0.0229**	1.0000			
DMARDs	0.1484 0.0594	0.0254 0.7483	0.0848 0.2833	0.0536 0.5130	−0.1246 0.1274	−0.1343 0.0884	0.0462 0.5592	1.0000		
TNFi	−0.0496 0.5312	−0.0099 0.9003	−0.0907 0.2510	−0.0530 0.5184	0.0410 0.6169	0.0347 0.6611	−0.1044 0.1861	**−0.2554** **0.0010**	1.0000	
Ln-RHI	−0.0244 0.7588	−0.0907 0.2527	**−0.1767** **0.0250**	0.1005 0.2197	**−0.1607** **0.0494**	−0.1211 0.1259	−0.0646 0.4182	−0.0368 0.6448	−0.0446 0.5771	1.0000

PSH, protein-SH; MDA, malondialdehyde; PON-1, paraoxonase-1; CRP, C-reactive protein; ESR, Erythrocyte Sedimentation Rate; DAS-28, Disease Activity Score-28; DMARDs, disease modifying antirheumatic drugs; TNFi, TNF-inhibitors; Ln-RHI, logarithmic reactive hyperaemia index; significant correlations are highlighted in bold.

**Table 5:** Independent determinants of Ln-RHI.

Independent Variable	Univariate Linear Regression B coefficient (95%IC), *p*	Multiple Linear Regression B Coefficient (95%IC), *p*
ESR	−0.002 (−0.004 to −6.040), 0.049	−0.003 (−0.005 to −0.0008), 0.008
MDA	−0.072 (−0.134 to −0.009), 0.025	−0.071 (−0.135 to −0.006), 0.032

A linear regression model with the ENTER method was calculated including age and gender as independent variables. ESR, erythrocyte sedimentation rate; MDA, malondialdehyde.

**Table 6:** Independent determinants of the presence of peripheral endothelial dysfunction (ED) in RA patients.

Model 1					
independent variables	Odds Ratio	SE	z	*p*	95%CI
MDA	1.751144	0.4472219	2.19	0.028	1.061536 to 2.888743
age	1.007933	0.0326649	0.24	0.807	0.9459022 to 1.074032
gender	1.223209	0.5648958	0.44	0.663	0.4947698 to 3.024113
ESR	1.021651	0.0092615	2.36	0.018	1.003659 to 1.039965
constant	0.0222555	0.0418738	−2.02	0.043	0.0005571 to 0.8891565
**Model 2**					
independent variables	Odds Ratio	SE	z	*p*	95%CI
PON-1	1.006696	0.002803	2.40	0.017	1.001217 to 1.012205
age	1.011697	0.0329131	0.36	0.721	0.949202 to 1.078306
gender	1.513167	0.7026411	0.89	0.372	0.6090183 to 3.759616
ESR	1.021511	0.0092764	2.34	0.019	1.003491 to 1.039855
constant	0.0337423	0.0609748	−1.88	0.061	0.0009772 to 1.165086

Odds ratio is based on the risk of peripheral ED. 95% CI, 95% confidence interval. SE, standard error. MDA, malondialdehyde. PON-1, paraoxonase-1.
